# A protective effect of morning radiotherapy on acute skin toxicity in patients with breast cancer: A prospective cohort study

**DOI:** 10.1097/MD.0000000000027155

**Published:** 2021-10-22

**Authors:** Marceila A. Fuzissaki, Carlos E. Paiva, Marco A. Oliveira, Marcelo A. Maia, Paula P.L. Canto, Yara C.P. Maia

**Affiliations:** a Medical School, Federal University of Uberlandia, Uberlandia, Minas Gerais, Brazil; b Department of Clinical Oncology, Division of Breast & Gynecology, Pio XII Foundation - Barretos Cancer Hospital, São Paulo, Brazil; c Center for Epidemiology and Biostatistics, Pio XII Foundation - Barretos Cancer Hospital, São Paulo, Brazil; d Faculty of Computing, Federal University of Uberlandia, Uberlandia, Minas Gerais, Brazil; e Department of Clinical Oncology, Clinics Hospital, Federal University of Uberlandia, Uberlandia, Minas Gerais, Brazil.

**Keywords:** breast neoplasms, chronotherapy, dermatitis, radiotherapy, risk factors

## Abstract

The focus of this prospective cohort study was to evaluate the risk factors of severe acute skin toxicity (grade ≥2) in 100 patients with breast cancer (BC) during radiotherapy (RT).

The patients were evaluated weekly during RT and 3 months after treatment. The endpoint included the occurrence of skin toxicity grade ≥2, according to Radiation Therapy Oncology Group (RTOG). Survival analysis was conducted by univariate and multivariate Cox regression analysis.

In the multivariate analysis, RT in the afternoon (0–3 pm) (hazard ratios [HR] = 1.566, *P* = .042) was significantly associated with the early occurrence of skin toxicity, indicating a potential effect of chronotherapy related to this adverse event. In the univariate and multivariate analysis, skin phototype moderate brown (HR = 1.586, *P* = .042; HR = 1.706, *P* = .022, respectively) and dark brown or black (HR = 4.517, *P* < .001; HR = 5.336, *P* < 0.001, respectively) was significantly associated with the skin toxicity. Tangential field separation >21 cm (HR = 2.550, *P* = .009, HR = 2.923, *P* = .003), in women that were submitted to conservative surgery indicates indirectly that large breast size was also significantly associated with skin toxicity.

Women with large breasts and dark brown or black skin should be followed more carefully during RT, which should be undergone in the morning, especially when submitted to conventional RT techniques, common in developing countries.

## 1 Introduction

Skin toxicity is one of the major adverse local events of radiotherapy (RT), with a negative impact on the quality of life in women with breast cancer (BC) and which may lead to interruption of the treatment.^[1,2]^ Although the risk factors for skin toxicities have been well reported in the scientific literature^[3–6]^ there is a shortage of data related to the influence of chronotherapy.^[7]^ Clinical studies have indicated that the time of day that patients undergo RT can significantly influence the response to treatment and the severity of toxicities ^[7–10]^ and chronotherapy may have a potential effect to reduce skin toxicity.^[11]^

Chronotherapy considers the influence circadian rhythms have on the different types of treatments.^[12]^ In mammals, circadian rhythms have predictable fluctuations over a 24-hour period that affect behavioral, biochemical and physiological processes. The master circadian marker is the suprachiasmatic nucleus (SCN) that drives rhythmic cycles within extra- SCN neurons and peripheral tissues, such as skin.^[13]^ One of the functions of the SCN is to direct the cell cycle progression.^[10,11]^ Each phase of the cell cycle corresponds to different degrees of radiosensitivity, with phase 2 and mitosis being the most radiosensitive, whereas the cells in the synthesis phase (S) are less sensitive to radiation. During treatment, radiation can damage normal cells that rapidly proliferate because of their high radiosensitivity, thus leading to adverse events. Such events can be minimized if the patient's treatment is performed during the time of day in which the non-neoplasic cells are in the S phase, that is, at which stage the cells are less sensitive to radiation.^[14]^

Therefore, the focus of this prospective cohort study was to predict the risk factors of this adverse event, including the time of day in which women performed RT. We hypothesized that among the risk factors identified, undergoing RT in the afternoon (0–3 pm) would be one of them.

## 2 Methods

This prospective cohort study was conducted with BC women during RT, from April 2016 to June 2017, at a University Hospital.

### 2.1 Recruitment strategy

Patients were selected from the daily list of the hospital and before starting their RT treatment and those that met the inclusion criteria were invited to participate.

### 2.2 Eligibility criteria

**Figure 1:**
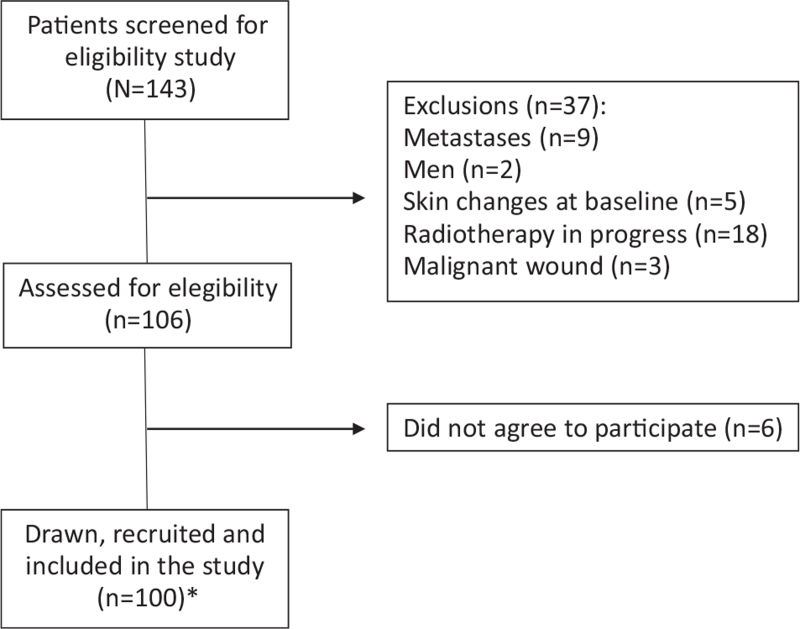
The number of women with breast cancer on radiotherapy included during the study, 2016-2017 (15). ^∗^100 patients were included in this study. However, 2 patients underwent radiotherapy for bilateral breast treatment, with an N = 102 irradiated sites for the analysis of skin toxicity.

The study involved women over the age of 18; any ethnicity; with diagnosis of non-metastatic BC; who underwent external RT. Those women who presented ulceration, a wound or skin tumor at the irradiation site; a history of hereditary diseases such as lupus erythematosus, rheumatoid arthritis, ataxia telangiectasia, who had already started their RT treatment and those women with a history of RT were excluded from the study.^[15]^ This selection process is illustrated in Flowchart 1 (Fig. 1).

### 2.3 Data collection

At first, on the day of RT planning, before starting the treatment, a semi-structured interview was carried out, based on a questionnaire developed by the researchers, in order to obtain data related to sociodemographic characteristics. Clinical and treatment characteristics were obtained by consulting the patients’ medical records.

Sequentially, the women were evaluated weekly during the RT and up to 3 months after the end of the treatment. It is noteworthy that, at all times, a dermatological physical examination was performed focusing on the irradiated area with photographic records.

### 2.4 Skin evaluation

Regarding the characteristics related to acute skin toxicity, the Radiation Therapy Oncology Group (RTOG) scale was used where

1. No change over baseline;
2. Follicular, faint or dull erythema/epilation/dry desquamation/decreased sweating;
3. Tender or bright erythema, patchy moist desquamation/moderate edema;
4. Confluent, moist desquamation other than skin folds, pitting edema;
5. Ulceration, hemorrhage, necrosis.^[16]^

The irradiated area was recorded, using a Canon EOS Rebel T5i 18 to 55 mm camera, with a resolution of 18 MP, aiming to reduce subjectivity in the evaluation. The photos were taken so that all possible sites for the occurrence of skin toxicity could be recorded and maximum care was taken to maintain confidentiality. The photos were evaluated by 3 expert professionals that completed a questionnaire with the RTOG scale. The final score was considered the one where there was agreement between 2 or 3 of the professionals. The photos that could not be agreed upon by the 3 expert professionals were reviewed and a final consensus was reached.^[16]^

### 2.5 Patient and treatment- related variables

The variables age, schooling, marital status, menopause (event considered after 1 year of amenorrhea according to the World Health Organization) were analyzed. The cutaneous phototype was graded according to the Fitzpatrick classification and divided into 3 categories as described by.^[15]^ The body mass index (BMI) was grouped into eutrophic (18.4 <BMI <25 for patients up to 64 years of age and 22 ≤BMI ≤27 for patients 65 years of age or older) and no eutrophic (BMI ≤18.4 or BMI ≥25 for patients up to 65 years of age and BMI <22 or >27 for patients aged 65 years or older).^[17,18]^

The number of nursing consultations during RT was grouped in greater than or equal to 5 consultations and less than or equal to 4. The reference for this grouping was that the patient should have the minimum number of 4 visits up to 21 days of treatment. Frequency of bra wear was grouped into 2 categories: not frequent, those that did not wear a bra during radiotherapy or used 1 for less than 2 weeks; and frequent, patients that wore bras for more than 2 weeks during radiotherapy.

Treatment variables such as chemotherapy and type of regimen, surgery, and endocrine therapy were analyzed.

### 2.6 Clinical variables

The pathological stage^[19]^ and molecular subtype^[20]^ were defined according to the literature.

### 2.7 Radiotherapy-related variables

Tangential field separation (breast width, in cm, at the posterior border of the medial and lateral tangential beams) was classified in accordance with the percentile (pc) (< pc 35: < 18 cm; pc 35–65: 18–21 cm; > pc 65: > 21 cm).^[21]^

Regarding the period of RT, the morning (07–10 am) and afternoon (0–3 pm) were considered. The number of fields of radiation has been grouped in 2 or more fields. Total dose was classified into 2 groups: less than 56  Gray (Gy); and greater than or equal to 56 Gy. Daily dose was also classified into 2 groups: 1.80 Gy and 2 Gy. The maximum radiation dose was defined according to the 75th percentile and grouped by greater than or equal to 110% and less than 110%.^[6]^

### 2.8 Ethical aspects and sample size calculation

This study was approved by the Human Research Ethics Committee (protocol number: 1348706/15) and was based on the standards of the Helsinki Declaration. All women signed a free and informed consent form and their privacy rights were observed.

The sample size required for this study was determined using the G∗Power software, version 3.1. The calculations were based on cox regression, fixed models, with expected effect size of 0.15, an alpha level of 0.05, 93% power. Given the output parameter, a total sample of 100 women was required at final analysis.^[15]^

### 2.9 Statistical analysis

Basic demographics, treatment and clinical characteristics of the cohort study were described using measures of central tendency and dispersion for continuous variables and proportions for categorical data.

Survival analysis was conducted using the Kaplan–Meier method, to identify the cumulative incidence and the possible differences in the curves for each exposure group. The occurrence of skin toxicity (grade ≥2 according to RTOG scale) was considered as an event.

The univariate and multivariate Cox regression analysis were used to predict the risk of developing skin toxicity in BC patients. The results were expressed as the relative probabilities of an event with 95% confidence intervals (CI). Variables with p values ≤0.2 were inserted into the multivariate Cox and the stepwise approach (with backward stepwise approach - wald method) was conducted. Values of *P* < .05 were considered statistically significant. Statistical analyzes were performed using SPSS software (IBM SPSS Statistics version 21).

## 3 Results

The total dose to the chest wall was 57.3 (95% CI 56.196–58.348) and a daily dose of 1.8 Gy in 37%, 2 Gy in 63%. 14% of the patients needed to interrupt treatment due to skin toxicity, with 7 days and 17 days being the minimum and maximum time. 73% received additional radiation dose, with 9 Gy (24%), 10 Gy, (48%), and 16 Gy (1%). The maximum treatment time was 50 days and the minimum was 27 days. The mean treatment time was 36.9 days (95% CI 35.70–37.88). The clinical and treatment characteristics are described in.^[15]^

**Figure 2:**
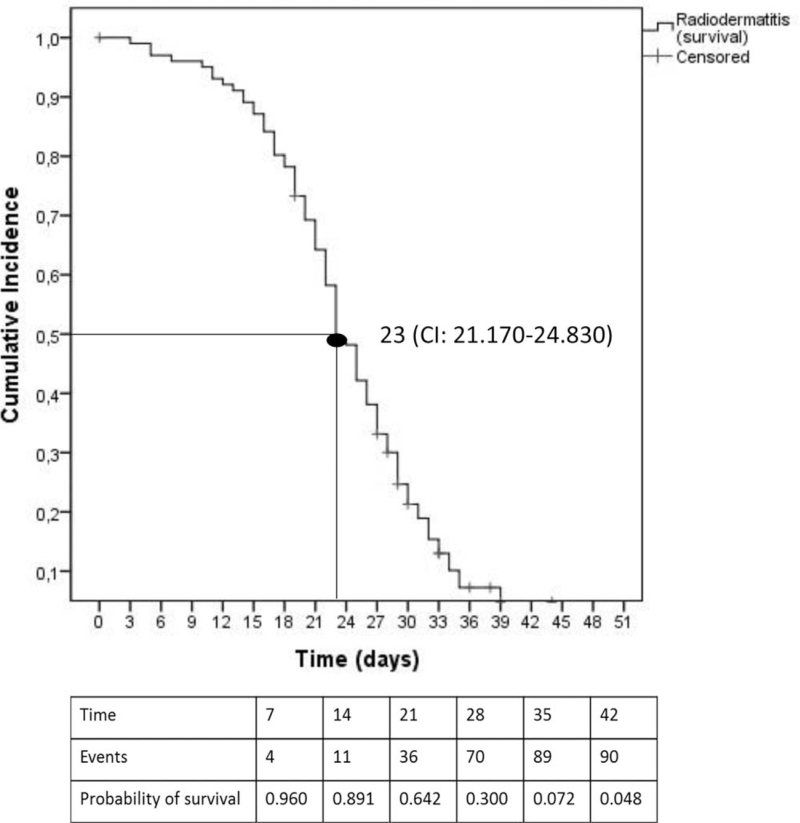
Conditional probability of skin toxicity in period of radiotherapy, according to Kaplan-Meier method. CI = confidence interval.

After 42 days, 88.2% (n = 90) of the patients presented skin toxicity. The median occurrence was after 23 days (95% CI 21.17–24.83), corresponding to a mean radiation dose of 38.85 Gy (95% IC 30.00–46.80) (Fig. 2).

Regarding skin toxicity (grade ≥2) risk factors, in the univariate analysis, maximum radiation dose ≥110% (hazard ratios [HR] = 1.637, *P* = .032), tangential field separation >21 cm (HR = 2.550, *P* = .009), phototype moderate brown (HR = 1.586, *P* = .042), dark or black (HR = 4.517, *P* < .001) and those who used a bra (for more than 2 weeks during RT) (HR = 1.633, *P* = .025) were significantly associated with the early occurrence of skin toxicity. In the multivariate analysis RT in the afternoon (HR = 1.566, *P* = .042), tangential field separation >21 cm (HR = 2.923, *P* = .003), moderate brown (HR = 1.706, *P* = .022), dark or black (HR = 5.336, *P* < .001) were significantly associated with the early occurrence of skin toxicity (Table 1).

**Table 1:** Risk factors on skin toxicity (grade ≥2) by Cox proportional-hazards model (n = 102).

			Univariate		Multivariate	
Variables	N total	N events	CRUDE HR (95% CI)	*P* value	ADJUSTED HR (95% CI)	*P* value
Age						
≥55 yr	55	48	1			
< 55 yr	47	42	1.271 (0.837–1.929)	.261		
Years of study						
<8 yr	56	51	1			
From 8 to 11 yr	11	10	0.764 (0.383–1.526)	.446		
>11 yr	35	29	1.083 (0.684–1.714)	.733		
Radiotherapy time						
Morning (7–10 am)	64	53	1		1	
Afternoon (0–3 pm)	38	37	1.363 (0.894–2.077)	.150	1.566 (1.017–2.411)	.042
Total dose of radiation						
<56 Gy	30	23	1			
≥56 Gy	72	67	1.338 (0.831–2.153)	.230		
Daily dose						
1.8 Gy	37	28	1			
2 Gy	65	62	1.224 (0.782–1.916)	.376		
Tangential field separation						
<18 cm (< pc 35)	18	14	1		1	
18–21 cm (pc 35–65)	62	56	1.403 (0.776–2.536)	.262	1.469 (0.809–2.665)	.206
>21 cm (> pc 65):	22	20	2.550 (1.267–5.135)	.009	2.923 (1.439–5.940)	.003
Number of RT fields						
2	43	38	1			
>2	59	52	1.122 (0.734–1.715)	.595		
Maximum dose of radiation						
<110%	70	61	1			
≥110%	32	29	1.637 (1.042–2.571)	.032		
Chemotherapy						
No	30	26	1			
Yes	72	64	1.251 (0.791–1.978)	.339		
Surgery						
Mastectomy	37	29	1			
Conservative Surgery	65	61	1.228 (0.786–1.917)	.366		
Hormone therapy						
No	32	28	1			
Yes	70	62	1.054 (0.674–1.650)	.817		
Phototype						
Type II or III	54	45	1		1	
Type IV	38	35	1.586 (1.016–2.476)	.042	1.706 (1.080–2.693)	.022
Type V or VI	10	10	4.517 (2.205–9.251)	<.001	5.336 (2.564–11.107)	<.001
Number of nursing consultations						
≤4	80	71	1			
≥5	22	19	1.015 (0.610–1.689)	.954		
Classification BMI						
Eutrophic	36	31	1			
No Eutrophic	66	59	1.538 (0.992–2.384)	.054		
Use of bra						
Not frequent	58	48	1			
Frequent	44	42	1.633 (1.062–2.510)	.025		

**Figure 3:**
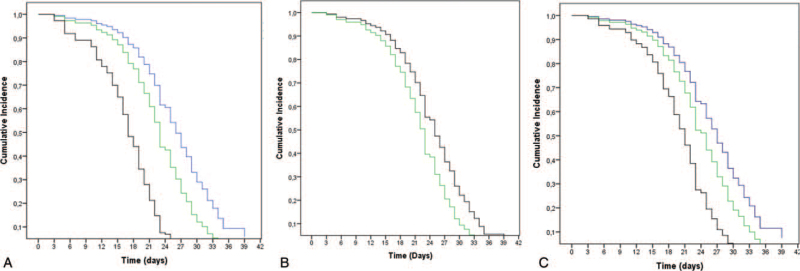
Cox Regression estimates of skin toxicity (grade ≥2). A) Fototype. In blue: Type II or III; Green: Type IV; Black: Type V or VI. B) RT period. In black: 7–10 am; Green: 12–3 pm. C) Tangential field separation. In blue: <18 cm; Green: 18–21 cm; Black: >21 cm.

Figure 3 shows the occurrence of skin toxicity during RT over time. Related to the cutaneous phototype, 100% (10 of 10) of type V or VI, 92% (35 of 38) of type IV and 83% (45 of 54) type II or III had skin toxicity, with all type V or VI presenting with this adverse event after 30 days of RT. Considering the RT time, 83% (53 of 64) of the patients who underwent RT in the morning and 97% (37 of 38) of the women who were treated in the afternoon, after 35 days of treatment, presented the expected event. Finally, regarding the tangential field separation, 91% (20 of 22) of the patients with >21 cm, 90% (56 of 62) of the patients between 18 to 21 cm, and 78% (14 of 18) of those with <18 cm presented skin toxicity. After 25 days of treatment, only 20% of the patients with >21 cm of separation had not presented skin toxicity.

## 4 Discussion

The main finding of the present study was that we observed a greater risk for the occurrence of skin toxicity in women who were treated in the afternoon when compared to those who underwent RT in the morning, showing an impact of chronotherapy during treatment and supporting our hypothesis.

Some studies have evaluated the role of chronotherapy in the oncology field ^[22,23]^ and specifically in RT relating it to the occurrence of various adverse events^[7,8,24,25]^ and general survival.^[9,10,12]^ However, the results diverge, with studies indicating a higher occurrence of toxicities due to RT at different times of the day, such as diarrhea^[8]^ and other intestinal complications,^[10]^ mucositis^[24,25]^ and^[7]^ even worse survival rates,^[12]^ reinforcing the need for further studies in this area.

The divergence in the results mentioned above may be due to a number of factors and among them the fact that the circadian rhythms controlling the cellular cycles may differ depending on the region of the body. One study compared the rate of deoxyribonucleic acid synthesis in 5 regions of the gastrointestinal tract in rats and found variation in the amplitude and peak time of deoxyribonucleic acid synthesis between regions.^[26]^ Another aspect that could influence is seasonal variations. Studies in rats observed circannual rhythms in the proliferation of intestinal cells, bone marrow and lymphoid organs.^[27,28]^ There is also the age-dependent effect on chronotherapy. Advanced age has been associated with interruptions in circadian rhythms that result in a remarkable reduction in melatonin production and a decrease in the proliferative capacity of endothelial progenitor cells. These help in restoring tissue during RT.^[29]^ It was observed that patients with prostate cancer aged 70 years or older who received radiation at night had a higher prevalence of late-onset toxicities.

In the present study, having large breasts (tangential field separation >21 cm) and being submitted to conservatory surgery, was a risk factor for the development of early skin toxicity in both univariate and multivariate analysis (HR = 2.550, *P* = .009, HR = 2.923, *P* = .003, respectively). Regarding BMI, non-eutrophic women presented a greater chance of developing skin toxicity (HR = 1.538, *P* = .054) in the univariate analysis, however without statistical significance. Obesity and having large breasts are considered risk factors for the occurrence of skin toxicity.^[4–6]^ Moody et al ^[30]^ suggests that more serious toxicities in patients with large breasts are related to localized hot spots and heterogenic dose of radiation, since such hot spots are related to the maximum prescribed dose. In the present study, women who were treated with a maximum dose greater than or equal to 110% had a risk of developing skin toxicity earlier (HR = 1.637, *P* = .032). One possible aspect may be related to the existence of a higher percentage of mammary adipose tissue.^[31]^ Irradiated adipose tissue is an important source of autotaxin secretion that produces lysophosphatidic acid.^[32]^ It promotes a vicious inflammatory cycle with nuclear factor kappa B activation, Cyclooxygenase 2 expression, and increased signaling by secretion of inflammatory cytokines, chemokines and growth factors, including transforming growth factor alpha ( alpha), platelet-derived growth factor and autotaxin.^[33]^ Such inflammatory response is associated with cutaneous toxicities.^[34]^

In our study, another factor that presented statistical significance for the occurrence of early skin toxicity was the phototype V or VI (HR = 4.517, *P* < .001; HR = 5.336, *P* < .001, univariate, multivariate respectively) when compared to phototype II, III, and IV. Our results are in agreement with the literature.^[3,4,35]^ The dark pigment that gives the dark coloration of black or dark brown skin is due to the presence of more eumelanin. The production of this type of melanin occurs when alpha- melanocyte stimulating hormone (alpha melanocyte stimulating hormone) binds to the melanocortin 1 receptor. The high frequency of the melanocortin 1 receptor Single Nucleotide Polymorphism, specifically mutations in the R160 W allele, was associated with the presence of severe acute skin toxicity,^[35]^ suggesting a relationship between dark skin color and the presence of this adverse event. Genetic and molecular markers may also explain this relationship. Blaszyk et al^[36]^ observed differences in the pattern of p53 mutations acquired by black women with breast cancer when compared to white women. This gene is related to the changes in the cell cycle due to ionizing radiation, with consequent modifications in the epithelial maturation process of this specific population.^[14]^

Finally, we also identified that within 23 days, 50% of patients submitted to RT had a probability of skin toxicity occurrence, that is, with a mean radiation dose corresponding to 38.85 Gy. Our data is similar to the literature,^[34,37]^ which also reports the occurrence of moist desquamation (corresponding to grade 2 or higher, according to the RTOG scale) in approximately 4 weeks or more (a radiation dose of between 30 and 40 Gy). Such data is extremely important, since it proves the need to adopt preventive measures and health education actions that have a significant impact before 23 days of treatment, that is, with an average radiation dose of approximately 40 Gy.

Despite advances in the field of RT, with the adoption of high-tech devices, providing more effectiveness and consequently a reduction of adverse events,^[38]^ conventional regimens without 3D planning is still a reality in developing countries such as Brazil. Thus, the results of the present study may contribute to public health care, and should be considered in the clinical practice of hospitals that follow the Brazilian Unified National Health System and other similar health care systems from developing countries.

Some limitations should be considered such as the small amount of variability in the sample included in the present study, specifically in relation to factors like smoking, chemotherapy, hormone therapy, radiotherapy scheme used. A secondary limitation is that it was not possible to evaluate patients undergoing more advanced RT techniques such as Intensity Modulated Radiotherapy. The strength of the study resides in its comprehensive nature and the quality of the data because symptoms were collected weekly during and also after the end of the radiation treatment. The evaluation of skin toxicity was realized to reduce subjectivity, through the photographic registry and independent evaluation by three expert professionals.

Considering that the period of the day when BC patients are treated is the only modifiable risk factor, it is suggested that those with large breasts and dark brown or black skin color, who had a higher risk of developing skin toxicity earlier, be treated in the morning (07–10 am), specifically those from developing countries, such as Brazil, which in many public hospitals still use conventional techniques.

## Author contributions

**Conceptualization:** Marceila de Andrade Fuzissaki, Paula Philbert Lajolo Canto, Yara Cristina de Paiva Maia.

**Data curation:** Marceila de Andrade Fuzissaki, Yara Cristina de Paiva Maia.

**Formal analysis:** Marceila de Andrade Fuzissaki, Carlos Eduardo Paiva, Marco Antonio Oliveira, Marcelo Almeida Maia, Yara Cristina de Paiva Maia.

**Funding acquisition:** Marcelo Almeida Maia, Yara Cristina de Paiva Maia.

**Investigation:** Marceila de Andrade Fuzissaki, Yara Cristina de Paiva Maia.

**Methodology:** Marceila de Andrade Fuzissaki, Paula Philbert Lajolo Canto, Yara Cristina de Paiva Maia.

**Project administration:** Marceila de Andrade Fuzissaki, Yara Cristina de Paiva Maia.

**Supervision:** Marceila de Andrade Fuzissaki, Yara Cristina de Paiva Maia.

**Validation:** Marceila de Andrade Fuzissaki, Yara Cristina de Paiva Maia.

**Visualization:** Marceila de Andrade Fuzissaki, Marcelo Almeida Maia, Paula Philbert Lajolo Canto, Yara Cristina de Paiva Maia.

**Writing – original draft:** Marceila de Andrade Fuzissaki, Carlos Eduardo Paiva, Marco Antonio Oliveira, Marcelo Almeida Maia, Paula Philbert Lajolo Canto, Yara Cristina de Paiva Maia.

**Writing – review & editing:** Marceila de Andrade Fuzissaki, Carlos Eduardo Paiva, Marco Antonio Oliveira, Marcelo Almeida Maia, Paula Philbert Lajolo Canto, Yara Cristina de Paiva Maia.
